# Genome-wide association studies and heritability analysis reveal the involvement of host genetics in the Japanese gut microbiota

**DOI:** 10.1038/s42003-020-01416-z

**Published:** 2020-11-18

**Authors:** Sachiko Ishida, Kumiko Kato, Masami Tanaka, Toshitaka Odamaki, Ryuichi Kubo, Eri Mitsuyama, Jin-zhong Xiao, Rui Yamaguchi, Satoshi Uematsu, Seiya Imoto, Satoru Miyano

**Affiliations:** 1 DeNA Life Science, Inc., Tokyo, Japan; 2 Morinaga Milk Industry Co., Ltd., Kanagawa, Japan; 3 Laboratory of DNA Information Analysis, Human Genome Center, The Institute of Medical Science, The University of Tokyo, Tokyo, Japan; 4 Department of Immunology and Genomics, Osaka City University Graduate School of Medicine, Osaka, Japan; 5 Division of Innate Immune Regulation, International Research and Development Center for Mucosal Vaccines, The Institute of Medical Science, The University of Tokyo, Tokyo, Japan; 6 Division of Metagenome Medicine, Human Genome Center, The Institute of Medical Science, The University of Tokyo, Tokyo, Japan; 7 Division of Health Medical Intelligence, Human Genome Center, The Institute of Medical Science, The University of Tokyo, Tokyo, Japan

**Keywords:** Microbiome, Genetic association study, Heritable quantitative trait

## Abstract

Numerous host extrinsic and intrinsic factors affect the gut microbiota composition, but their cumulative effects do not sufficiently explain the variation in the microbiota, suggesting contributions of missing factors. The Japanese population possesses homogeneous genetic features suitable for genome-wide association study (GWAS). Here, we performed GWASs for human gut microbiota using 1068 healthy Japanese adults. To precisely evaluate genetic effects, we corrected for the impacts of numerous host extrinsic and demographic factors by introducing them as covariates, enabling us to discover five loci significantly associated with microbiome diversity measures: *HS3ST4*, *C2CD2*, 2p16.1, 10p15.1, and 18q12.2. Nevertheless, these five variants explain only a small fraction of the variation in the gut microbiota. We subsequently investigated the heritability of each of the 21 core genera and found that the abundances of six genera are heritable. We propose that the gut microbiota composition is affected by a highly polygenic architecture rather than several strongly associated variants in the Japanese population.

## Abstract (web summary)

Ishida et al. investigated genome-wide associations between 1068 healthy Japanese adults and their gut microbiota. They find that five loci account for a small variation of the human gut microbiota composition and that six genera are heritable. This study suggests that human gut microbiota composition is likely to be affected by multiple genetic loci.

## Introduction

Recently, interest in the gut microbiome has increased because of its tremendous potential to impact our physiology^1^. A significant portion of previous research has focused on discovering links between bacterial composition and various diseases such as metabolic syndromes, cancers and inflammatory bowel disease (IBD), while a fundamental understanding of factors shaping the gut microbiota composition of healthy individuals remains to be elucidated. Therefore, a major concern is how various types of host intrinsic and extrinsic factors contribute to the microbiota composition. Numerous host extrinsic factors, including diet, medication, physical activity, and health status, have been reported to affect the microbiota composition^2–5^. Conversely, the contribution of host genetics has only recently emerged and only a limited number of studies have been reported. In human studies, Goodrich et al. demonstrated that microbial communities in monozygotic twins were more similar than those in dizygotic twins, indicating that gut microbial composition was influenced by host genetics^6^. Previous studies have identified several heritable bacterial taxa, including *Faecalibacterium*, Unclassified Ruminococcaceae (Ruminococcaceae;g), *Coprococcus*, *Bifidobacterium*, *Parabacteroides*, and *Bacteroides*, using European and Korean populations^7–11^. The first genome-wide association study (GWAS) using 93 subjects from the Human Microbiome Project (HMP) identified several key host genes affecting the microbiota, including the *LCT* gene, whose variant correlated with the abundance of *Bifidobacterium*^12^. The effect of the *LCT* gene variant has been replicated in multiple studies using more than 1000 individuals with European ancestry, showing indubitable evidence^5,8,10,13,14^. Our previous targeted approach also indicated a possible contribution of monomorphic *LCT* gene variants associated with low lactase activity to the high abundance of *Bifidobacterium* in Japanese subjects^15^. However, genome-wide approaches to study the human gut microbiota have been restricted to Europeans, and the genomic basis for the microbiota in other populations remains unclear since genetic backgrounds differ among populations. Furthermore, most of the previous GWASs did not comprehensively consider numerous extrinsic factors for the precise evaluation of genetic factors.

Japanese people have a characteristic gut microbial composition compared to those of people from other nations that cannot be simply explained by differences in dietary habits alone^16^. In addition, the Japanese population has often been viewed as an Asian population with a more genetically homogeneous community than other populations such as Europeans^17^. This homogeneity is a suitable feature for GWAS because this approach assumes a homogeneous population in which the relationship between a SNP and a phenotype is random under the null hypothesis, and the presence of population stratification can cause spurious associations. Therefore, the GWAS analysis of the Japanese gut microbiota may reveal novel findings similar to those obtained from previous GWASs of several phenotypes^18,19^.

This study reports the application of GWAS to identify associated loci related to Japanese gut microbiota for the first time to the best of our knowledge using 1068 healthy Japanese adults recruited from the customer base of MYCODE, a personal genome service in Japan. We incorporated 138 extrinsic and demographic variables as covariates for the analysis, which allowed us to precisely evaluate genetic associations. We also estimated the heritability of abundances of individual microbial genera to reveal a total contribution of common genetic factors.

## Results

### Characterization of Japanese gut microbiota

We utilized 16S rRNA gene sequencing to characterize the gut microbiota of 1068 healthy Japanese adults, whose background data are shown in Table 1. A total of 9,285,977 high-quality paired sequences were obtained from fecal samples, and 8695 ± 2255 (mean ± standard deviation) reads per sample were generated. We focused on the relative abundances of the 21 core genera found in more than 95% of the Japanese subjects among the numerous bacterial groups, the alpha diversity, including Chao1, Shannon, and phylogenetic diversity (PD whole tree) (Supplementary Data 1), and the beta diversity based on weighted UniFrac distance. The median (25th–75th percentiles) of the total relative abundance of the core 21 genera was 89.4% (83.7–92.8%).

**Table 1:** Background of participants.

Characteristics	Values
Total no.	1068
Female (%)	50.7
Age (years)	41 [34, 48]
BMI (kg/m^2^)	21.42 [19.74, 23.68]

For the age and BMI, the median and the interquartile range [25th quartile, 75th quartile] are listed.

### Associations of extrinsic and demographic factors with gut microbial composition

To identify candidate covariates for GWAS analysis, we investigated the associations of various host extrinsic and demographic factors with gut microbiota parameters, the relative abundances of the 21 core genera and the bacterial diversity indices. A total of 138 variables were composed of nutrition (43 variables), food (74 variables), and nondietary groups (21 variables), which were sub-classified into five categories (demographics, physical characteristics, lifestyle, stress response, and health information) (Supplementary Data 2). A total of 281 significant associations of those variables were observed when compared with the relative abundances of the 21 bacterial groups (Supplementary Data 3) and the bacterial diversities (Supplementary Data 4), although 257 (91%) of their absolute coefficients values were less than 0.2, indicating weak effects. For the beta diversity, no significant association was observed.

**Fig. 1:**
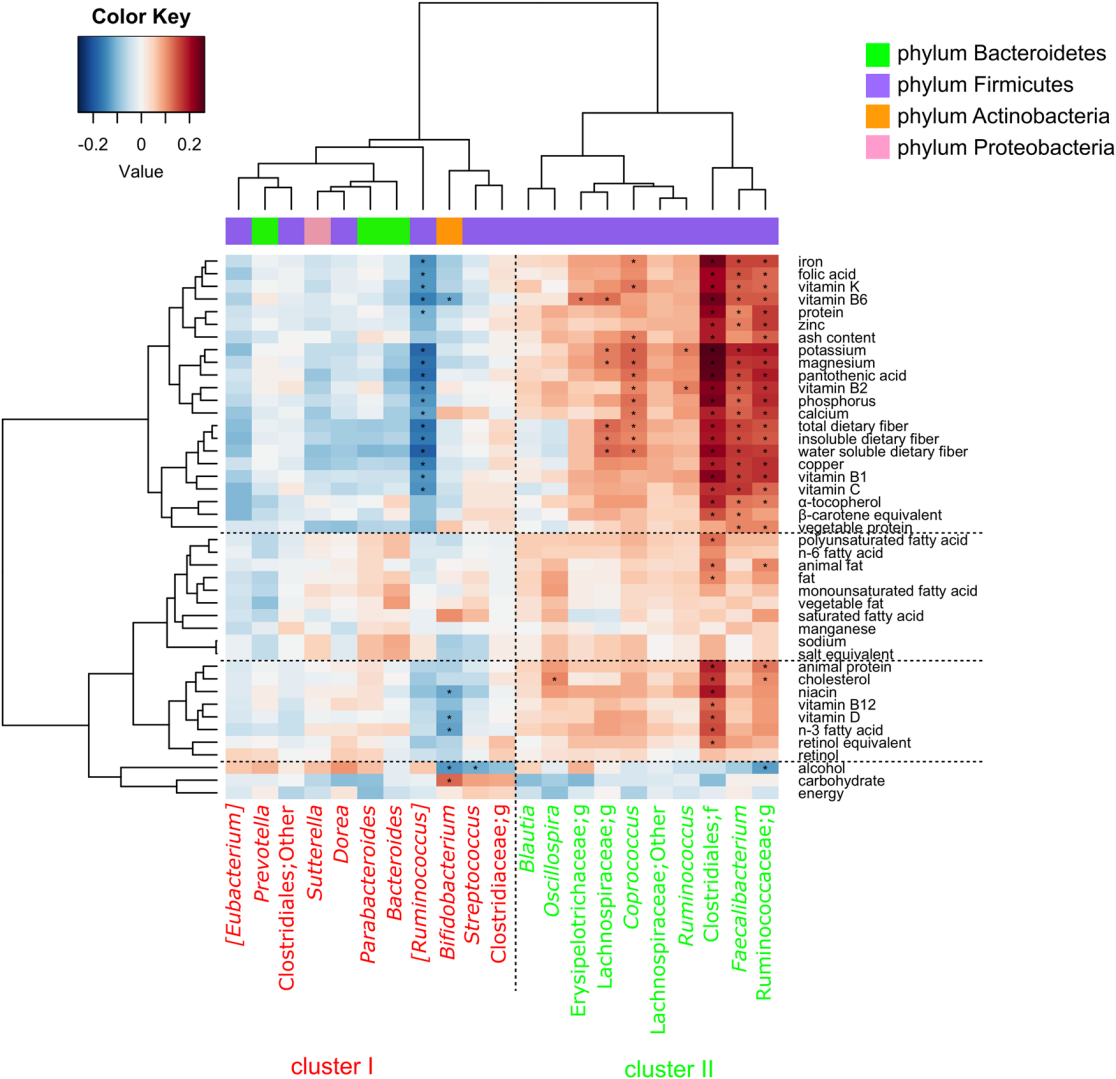
A hierarchical clustering of the 21 core genera based on association with nutrition intake. Columns correspond to the 21 core genera in the Japanese gut; rows correspond to nutrition intake measured by BDHQ. Red and blue denote positive and negative associations, respectively. The intensity of the colors represents the degree of association between the genus abundance and nutrition intake as measured by multiple linear regression analysis. Bacterial phyla are summarized by the color code on the top right. The dots indicate the associations that are significant after adjusting for multiple testing of 138 variables. Columns and rows are subjected to hierarchical clustering. The genera in cluster I are indicated in red, and those in cluster II are indicated in green. All regression coefficient values are listed in Supplementary Data 5.

Subsequently, we performed clustering of the 21 core genera based on the regression coefficient values of nutrition (Fig. 1 and Supplementary Data 5), food (Supplementary Fig. 1) and nondietary variables (Supplementary Fig. 2). The bacterial groups were divided into two major clusters, “cluster I” and “cluster II”, based on nutrition data (Fig. 1). Interestingly, the clustering patterns based on nutrient/food intake were similar to those based on nondietary variables, and the trend was significant (Fisher’s exact test, *P* = 0.00387). *Bifidobacterium*, which is a well-known beneficial genus, showed positive associations with the intake amount of carbohydrates and full-fat milk, which contains lactose (Supplementary Data 3 and Fig. 1 and Supplementary Fig. 1). These data are consistent with those of previous studies that demonstrate the superiority of *Bifidobacterium* for carbohydrate utilization^2,20,21^.

Given the detected associations of the extrinsic and demographic variables with the gut microbiota, we introduced these variables as covariates in the subsequent host genetic variants versus microbiome association analyses.

### The influence of host genetic loci on the gut microbiota

**Fig. 2:**
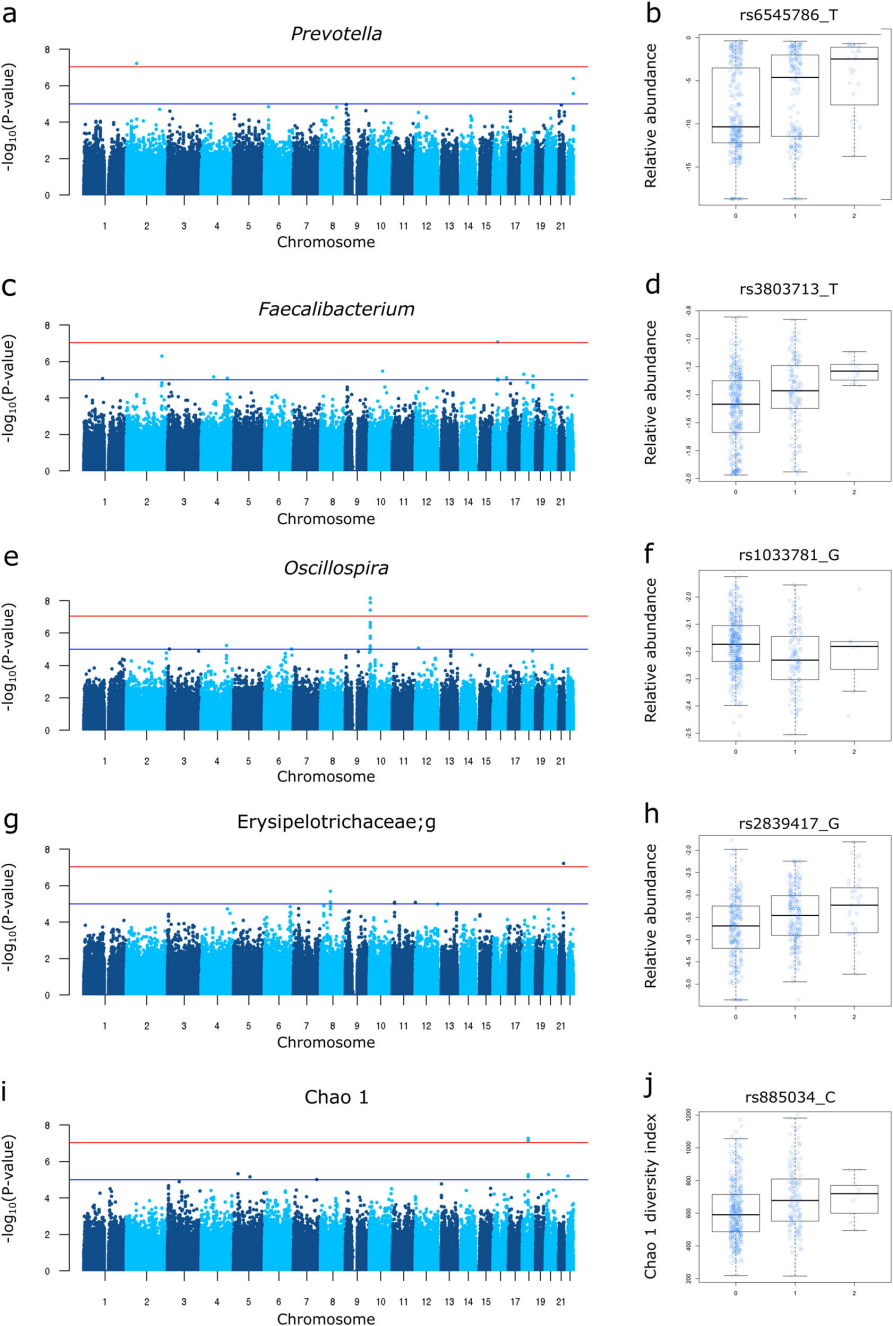
Significant associations of the genetic loci for the gut microbiota. **a**, **c**, **e**, **g**, **i** Manhattan plots of (**a**) *Prevotella* in males, (**c**) *Faecalibacterium* in males, (**e**) *Oscillospira* in females, (**g**) Erysipelotrichaceae;g in females, and (**i**) the diversity index Chao1 in females under the additive model for (**a**, **c**, **g**) and the dominant model for (**e**, **i**). **b**, **d**, **f,** **h**, **j** Box plots of (**b**) *Prevotella* in males, (**d**) *Faecalibacterium* in males, (**f**) *Oscillospira* in females, (**h**) Erysipelotrichaceae;g in females, and (**j**) the diversity index Chao1 in females. The red line represents a genome-wide significance level (*P* = 8.9 × 10^−8^), and the blue line represents a genome-wide suggestive level (*P* = 1 × 10^−5^). In each box plot, the *x*-axis indicates the minor allele count of the SNP indicated on the top, and the *y*-axis represents the Box–Cox–transformed relative abundances of the genera (**b**, **d**, **f**, **h**) and the alpha diversity index Chao1 (**j**). Descriptive statistics are listed in Supplementary Data 8.

We employed GWAS to identify associated genetic loci for Japanese gut microbiota incorporating the host extrinsic and demographic factors as covariates, which are indicated in bold in Supplementary Data 3 and 4. We found a total of 108 loci that were suggestively associated (*P* < 1 × 10^−5^) with host microbiota parameters (Supplementary Data 6), but none of them reached the genome-wide significance level of *P* < 8.9 × 10^−8^, which was set by adjusting the number of SNPs tested per microbiota parameter on the basis of the Bonferroni method. Because of the reported sex differences in the gut microbiota^22–25^, we further performed a sex-stratified GWAS. Of the 195 suggestive associations found (Supplementary Data 7), five loci whose associations have never been reported before in European GWASs were significant (Table 2 and Supplementary Data 8). We further performed the imputation of the missing genotypes for these five loci, and their regional plots are shown in Supplementary Fig. 3. The abundance of *Prevotella* was associated with the 2p16.1 locus in male subjects (rs6545786, *P* = 5.93 × 10^−8^) (Fig. 2a, b, Supplementary Fig. 3a). The inclusion of covariates slightly reduced the association *p*-value of the loci (*P* = 7.01 × 10^−8^; without covariate analysis), showing a nominally significant effect of Bristol stool scale (*P* = 1.13 × 10^−4^). The *HS3ST4* locus was associated with the abundance of *Faecalibacterium* (rs3803713, *P* = 8.29 × 10^−8^) in male subjects (Fig. 2c, d, Supplementary Fig. 3b). The association *p*-value slightly decreased with the incorporation of covariates (*P* = 8.77 × 10^−8^; without covariate analysis), and three variables: age, “soup consumed with noodles”, and potassium showed nominally significant effects (*P* = 8.01 × 10^−3^, 1.34 × 10^−2^, and 2.07 × 10^−2^, respectively). The abundance of *Oscillospira* was associated with the 10p15.1 locus in female subjects (rs1033781, *P* = 5.28 × 10^−8^) and showed a lower *p*-value under a dominant model (*P* = 1.28 × 10^−8^) (Fig. 2e, f, Supplementary Fig. 3c). This significant association could be obtained only with the inclusion of covariates (*P* = 3.21 × 10^−7^; without covariate analysis), and Bristol stool scale, bowel movement frequency, and coffee variables showed nominally significant effects (*P* = 3.85 × 10^−17^, 2.22 × 10^−5^, and 4.71 × 10^−3^, respectively). The *C2CD2* gene locus was associated with the abundance of Erysipelotrichaceae;g (rs2839417, *P* = 5.96 × 10^−8^) in female subjects (Fig. 2g, h, Supplementary Fig. 3d). This association was not significant when the covariates were not incorporated (*P* = 4.10 × 10^−7^). “Rice cakes and Japanese-style pancakes”, the second principal component of the host genotypic data, and age variables showed nominal significant effects (*P* = 2.72 × 10^−3^, 4.12 × 10^−3^, and 2.23 × 10^−2^, respectively). The alpha diversity index, Chao1 showed a significant association with the 18q12.2 locus, which was located in a large intergenic region, in female subjects (rs885034, *P* = 8.24 × 10^−8^), and the association *p*-value decreased slightly when the dominant model was applied (*P* = 5.12 × 10^−8^) (Fig. 2i, j, Supplementary Fig. 3e). This significant association could be obtained only with the inclusion of covariates (*P* = 1.20 × 10^−7^; without covariate analysis), and three variables: “Rice cakes and Japanese-style pancakes”, the second principal component of the host genotypic data, and age showed nominal significant effects (*P* = 2.72 × 10^−3^, 4.12 × 10^−3^, and 2.23 × 10^−2^, respectively). The five newly identified loci showed significant sex differences in their effect sizes even though effect directions were the same in both sexes (Supplementary Data 9). In total, the proportion of the variance in each microbial parameter explained by individual SNPs was small, ranging from 1.1 to 2.0% (Table 2), consistent with a previous report in which the genetic influence on beta diversity indices was studied^13^. These results suggest that there is no SNP that strongly impacts the Japanese gut microbiota.

**Table 2:** Genome-wide significant SNPs associated with the microbiota parameters.

Bacterial group/diversity index	Sample	Locus	SNP	Chr	Position^a^	Minor/major allele	MAF^b^	Effect size (SE)^c^	The proportion of variance explained	*P*-value	Protein expression level in the small intestine (glandular cells)^f^	Protein expression level in the colon (endothelial cells)^f^	Protein expression level in the colon (glandular cells)^f^	Protein expression level in the colon (peripheral nerve/ganglion)^f^
Relative abundance														
* Prevotella*	Male	2p16.1	rs6545786	2	60403018	T/C	0.23	2.015 (0.366)	0.018	5.93E−08				
* Faecalibacterium*	Male	*HS3ST4*	rs3803713	16	25838494	T/C	0.15	0.111 (0.020)	0.013	8.29E−08				
* Oscillospira*	Female	10p15.1	rs1033781	10	4331114	G/T	0.14	−0.047 (0.008)	0.011	5.28E−08				
Erysipelotrichaceae;g	Female	*C2CD2*	rs2839417	21	43319737	G/A	0.28	0.250 (0.045)	0.020	5.96E−08	++	+	++	++
Alpha diversity index														
Chao1	Female	18q12.2	rs885034	18	36001573	C/A	0.15	78.19 (14.37)	0.012	8.24E−08				

^a^Positions were derived from dbSNP build 137.

^b^The minor allele frequency calculated using the data from all subjects.

^c^Linear regression coefficient beta and standard error (SE) of the minor allele.

^d^The proportion of variance explained by individual SNPs calculated based on the effect of a minor allele on the standardized trait

^e^SNPs were annotated with SNPnexus (http://snp-nexus.org/index.html).

^f^Protein expression levels of overlapped genes in intestinal and colonic tissues according to The Human Protein Atlas (http://www.proteinatlas.org/). +++: High, ++: Medium, +: Low, –: Not detected.

*Chr* chromosome, *SNP* single nucleotide polymorphism, *MAF* minor allele frequency.

Among a total of 303 suggestive associations identified in this study, only one association had already been reported in a European GWAS^5^, which indicated that an *ATXN1* gene region associates with the abundance of *Ruminococcus*. However, we obtained the association only in female subjects (rs729770, *P* = 2.35 × 10^−6^) (Supplementary Data 7), while the previous study did not distinguish male and female samples. Subsequently, we evaluated the effects of the previously reported gut microbiota-associated loci on the Japanese gut microbiota. Among our 219 typing SNPs selected from previous studies, including proxies of previously reported lead SNPs (Supplementary Data 10), congruent results were observed only for three SNPs, rs10485717, rs2423278, and rs2423279, which were proxies of rs6086208. They showed significant associations with the Chao1 index as reported in the previous European GWAS^9^ (Supplementary Data 11). The multiple previous European GWASs have demonstrated significant associations between the abundance of *Bifidobacterium* and the *LCT* gene SNPs, which are associated with lactase persistence owing to modulation of lactase enzymatic activity^5,8,10,12–14^. We analyzed the association by eight SNPs near the *LCT* gene (Supplementary Data 10). One was rs4988235, the potentially causal SNP, four were rs1050115, rs2304371, rs3754689, and rs6730157, which were in moderate or high linkage disequilibrium (LD) with rs4988235 in Europeans (r2 = 0.249, 0.375, 0.313, and 0.751, respectively, in the HapMap CEU data), and three were proxies of rs2164210 which was also in moderate LD with rs4988235 in Europeans (r2 = 0.662 in the HapMap CEU data). However, no SNP showed a significant association with the abundance of *Bifidobacterium*, and rs4988235 was not polymorphic in the Japanese population^15^.

### SNP heritability of gut microbiota

**Fig. 3:**
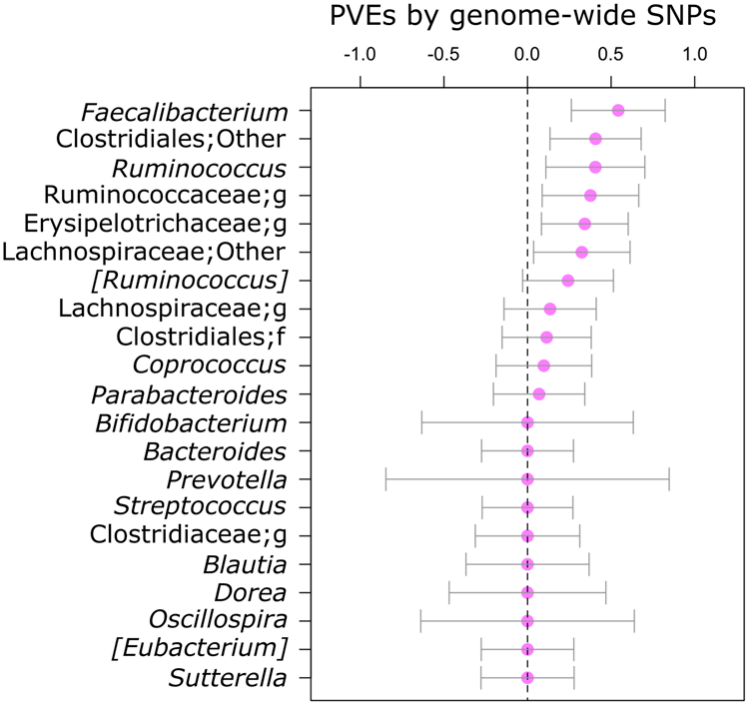
SNP heritability of the 21 core genera. Each point represents the estimated PVE by genome-wide SNPs for the abundance of the 21 core genera. Bars indicate SE measurements around the estimate. The posterior samples of PVE, which were obtained using the Markov chain Monte Carlo-based method in GEMMA, are plotted in Supplementary Fig. 4.

Even though individual common genetic variants did not exhibit strong impacts, their cumulative effects could contribute to the gut microbiota composition. Thus, we investigated the cumulative effects of multiple SNPs on the gut microbiota parameters. To this end, we estimated SNP heritability by calculating the percentage of variance explained (PVEs) by genome-wide SNPs using GEMMA^26^ on sex-combined data. As shown in Fig. 3 and Supplementary Data 12, the error bars of PVEs calculated against the abundances of six genera, *Faecalibacterium*, Clostridiales;Other, *Ruminococcus*, Ruminococcaceae;g, Erysipelotrichaceae;g, and Lachnospiraceae;Other, do not intersect at zero, indicating SNP heritability. When these six heritable genera were compared with the other 15 non-heritable genera, no differences in the combined effect sizes of suggestively associated SNPs, which were calculated by the predictive power (the adjusted R-squared value) of a multiple regression model incorporating lead SNPs we identified, were observed (Supplementary Fig. 5 and Supplementary Data 13). This result suggests that the Japanese gut microbiota composition is affected by multiple genetic variants whose individual effects are too small to reach a genome-wide suggestive level. We also studied replications of 14 taxa (five of which were included in the 21 core genera) which had nominally significant heritability estimates in at least two of four previous studies^10^ and were found in at least 50% of our samples. As a result, only two taxa were found to be heritable in our Japanese samples (Supplementary Data 14). One taxon was the *Faecalibacterium*, which was already described above, and the other taxon was the family Erysipelotrichaceae. Notably, the heritability estimate of the family Christensenellaceae reflected zero heritability in this study (Supplementary Data 14), despite this family has been reported to be heritable taxon in multiple previous studies^6,8,9,11^.

## Discussion

The gut microbiota composition shows large inter-individual variations, and identification of the factors contributing to gut microbial variations has been a major concern because previously identified factors could not sufficiently explain the variations. In this study, we performed the first GWAS for Japanese gut microbiota to the best of our knowledge using 1068 healthy Japanese adults to identify genetic loci related to the microbiota. We also investigated the heritability of Japanese gut microbiota using genome-wide SNPs.

A total of 303 genome-wide suggestive associations were identified for the Japanese gut microbiota, and five of them were loci significantly associated with the relative abundance of *Prevotella*, *Faecalibacterium*, *Oscillospira*, and Erysipelotrichaceae;g and the bacterial diversity Chao1 index (Table 2, Fig. 2). The *HS3ST4* gene, whose locus was related to *Faecalibacterium* abundance, encodes the enzyme heparan sulfate D-glucosaminyl 3-O-sulfotransferase 4, which transfers a sulfate group to the 3-OH position of N-acetylglucosamine (GlcNAc). The protein expression of the gene has not yet been examined in human tissues (Table 2), and its mRNA expression is reported to be enriched in the brain but hardly detectable in intestinal and colonic tissues (Genome-Tissue Expression (GTEx) project, http://www.gtexportal.org/home). The mechanism underlying the relationship between the *HS3ST4* gene variant and the gut microbiota should be further investigated. C2 domain-containing protein 2 encoded by the *C2CD2* gene is expressed in the intestine and colon^27,28^ (Table 2); however, its function remains unknown. A recent GWAS for colorectal cancer demonstrated that the *C2CD2* gene SNP was associated with advanced colorectal adenomas^29^, suggesting some function of the gene in the colon. The bacterial family Erysipelotrichaceae, one genus of which was found to be associated with the *C2CD2* gene SNP in our study, has been known as a colitogenic strain due to its enrichment in colorectal cancer in a human study and animal model studies^30–32^, suggesting that the *C2CD2* gene mediates the tumorigenesis of colorectal cancer through gut microbiota. The roles of the other three loci, located in intergenic regions, in the mechanism of shaping microbial composition remain unclear. The impact of individual associated SNPs was trivial and unable to sufficiently explain the variance in the gut microbiota parameters, although at least six genera were identified as heritable. From these results, we propose that no common genetic variant is strongly associated with the Japanese gut microbiota, and that the Japanese gut microbiota composition is a highly polygenic trait. The polygenicity of the human gut microbiota was proposed in several previous European studies demonstrating limited effects of specific SNPs^5,13,33^. Our findings represent that polygenic characteristics of the human gut microbiota are shared across European and non-European populations and reinforce the notion that genetic backgrounds should be comprehensively considered in future studies concerning the effects of human gut microbiota on host physiology.

The five loci identified in this study were not reported in previous European GWASs, and most reported associations from previous European GWASs were not replicated in our samples. These results indicate that population-specific genetics might affect the difference in the effect for each gut microbe. For one possible explanation, the different effect sizes of the SNPs might be due to the different gene-environment interactions or the difference in LD structure in the region between the two populations. Another possibility could be the low statistical power in the European population due to the low minor allele frequencies of the SNPs, such as rs1033781 (in the 10p15.1 locus), whose minor allele frequency is 0.004 in the HapMap CEU population. The *LCT* gene variants show correlations with the abundance of the genus *Bifidobacterium* in Europeans but not in Japanese people probably due to a monomorphic feature of the potentially causal SNP, rs4988235, in the Japanese population^15^. These different contributions of the *LCT* gene to the gut microbiota between the two populations support population-specific genetic effects. The *LCT* is reportedly one of the genes that has experienced extensive natural selection in Europeans^34^, and such natural selection would be one of the factors causing population-specific SNPs, leading to population-specific genetic effects. However, similar to initial GWASs for other complex traits, published GWASs of the human gut microbiota have shown little overlap in the associated loci even among the same European studies^10,33,35,36^. This discordance could be attributed to technical differences in microbiome data analyses, as well as a poor statistical power due to smaller sample sizes in human gut microbiome GWASs (in the low thousands) than the current GWAS standards (tens or hundreds of thousands). We should note here the following concerns in our study. One concern is that we used a more liberal threshold to extensively identify potential associations rather than a strict study-wide Bonferroni threshold, which was adjusted by not only the number of SNPs but also the number of phenotypes tested. The five loci identified with the threshold reached neither a strict study-wide Bonferroni threshold nor the usual genome-wide significance threshold of 5 × 10^−8^. The other concern is that our study lacked replications of the identified associations using independent cohorts because Japanese samples on a scale of a thousand are not currently available. These concerns may increase the false positive rate on the identified variants, and we cannot exclude the possibility that the above mentioned factors other than the population-specific genetics affected our results. A larger-scale analysis is required to confirm the population-specific genetic effects.

The five associations were significant only in the sex-stratified analysis, and significant sex differences were detected in their effects. The sex-specific differences in the gut microbiota were reported in multiple previous studies^22–25^. Sex hormone-related enzymes are already known to affect gut microbial differences between males and females^22,23^. Such sexual dimorphism in gene expression would explain sex-specific genetic effects. Thus, the sex-specific effects of the five loci should undergo further validation in independent cohorts.

We found four previous studies reporting heritability estimates, one of which studied Koreans, and the other three of which studied Europeans^7–11^. The most highly heritable genus in this study, *Faecalibacterium*, was also concluded to be heritable in these studies except for one European study^8–11^. For the second and third most highly heritable genera in our study, Clostridiales;Other and *Ruminococcus*, only one European study but not the other three agreed with our results^9^. The fourth most highly heritable genus in our study, Ruminococcaceae;g, was explored in only one European study, which concluded that it was heritable^8^. Concerning the other heritable genera in this study, Erysipelotrichaceae;g and Lachnospiraceae;Other, none of the four studies found significant heritability. Furthermore, 13 of the 15 heritable taxa reported previously did not show evidence of heritability in our study. Taken together, these results allow us to argue population-specific heritability, which would be caused by population-specific factors such as allele frequencies and variation in environmental conditions, in addition to heritability overriding population differences. However, we need more powerful studies to determine whether different conclusions for heritability estimates across studies result from population differences or other factors.

We demonstrated that combinatorial contributions of multiple common genetic variants affect the Japanese gut microbiota. However, our study design did not possess an efficient power to identify all the contributing loci. In addition, contributions of missing factors, such as rare genetic variants and gene-environment interactions, remain to be identified. To this end, we need to increase the number of samples. An open microbial consortium termed the MiBioGen consortium was recently initiated^36^. This consortium comprises 18 population-level worldwide cohorts for whom the gut microbiota, host genotype, and anthropometric, metabolic and disease-related individual outcomes, have been analyzed. This type of data set enables large-scale analyses and meta-analyses of diverse populations, which will generate new knowledge concerning the universal and population-specific effects of genetic factors on human gut microbiota.

## Methods

### Subjects

Healthy Japanese participants were selected for this study in the following manner. After purchase of MYCODE (DeNA Life Science Inc., Tokyo, Japan), a personal genome service in Japan, the customers sent back their saliva samples for genetic testing to the DeNA Life Science’s (DLS’s) laboratory with a written application form. On the application form, they can choose whether to give consent to participate in MYCODE Research, in which their anonymized genetic data and/or health-related information would be used for scientific research purposes.

Among participants in MYCODE Research, people between 20 and 64 years old were invited to participate in the specific research for this study by an email informing its overview. Potential participants expressing their interest provided informed consent for this study and completed a screening questionnaire online under a protocol approved by the ethics committee of DLS Inc. (protocol #20160727_1) and the ethical committee of Institute of Medical Science, the University of Tokyo (Tokyo, Japan) (IMSUT-IRB) (protocol #29-29-1125). In total, 1,907 people applied for the study over a four-day period, and 1083 turned out to be eligible for inclusion. The final 1068 subjects were selected through the quality control of subjects as described below, as well as in our previous study^15^.

### Genotyping and quality control

Subjects were genotyped on the Infinium OmniExpress-24+ BeadChip or Human OmniExpress-24+ BeadChip (Illumina Inc., San Diego, CA, United States). All of these experiments were performed at the DLS laboratory. Quality control procedures for the genotype data included the exclusion of SNPs with a minor allele frequency under 0.01, a Hardy-Weinberg equilibrium *p*-value below 10^−6^, or a call rate under 95%. The quality control of subjects was performed using the following criteria. First, two subjects with call rates under 95% were excluded. Subsequently, nine subjects who were one of a pair with a proportion of identical by descent greater than 0.185 and one subject failing a sex check were removed.

Principal component analysis (PCA) was performed based on the genotype data of the subjects after the quality control procedures described above using PLINK version 1.9^37,38^. Three outlier subjects were removed from the analysis based on visual inspection of the top two principal components. Then, we confirmed that all the residual subjects had Japanese ancestry by PCA using their data along with the Phase 3 HapMap data (ASW, African ancestry in Southwest USA; CEU, Utah residents with Northern and Western European ancestry from the CEPH collection; CHB, Han Chinese in Beijing, China; CHD, Chinese in Metropolitan Denver, Colorado; GIH, Gujarati Indians in Houston, Texas; JPT, Japanese in Tokyo, Japan; LWK, Luhya in Webuye, Kenya; MXL, Mexican ancestry in Los Angeles, California; MKK, Maasai in Kinyawa, Kenya; TSI, Toscani in Italia; YRI, Yoruba in Ibadan, Nigeria) (Supplementary Fig. 6). The final data set included 1068 subjects for 558,583 SNPs.

### Screening survey questionnaire

The screening survey questionnaire contained questions to select eligible subjects according to the inclusion and exclusion criteria described above, as well as to ask bowel movement frequency, yogurt intake frequency, and whether the subjects took supplements containing probiotics, oligosaccharide, or fiber more than twice a week. Five variables for the analysis were obtained from certain questions, which are listed in Supplementary Data 2.

### Fecal sampling and health conditions questionnaire

Subjects were provided with a fecal sample collection kit (Techno Suruga Laboratory Co., Ltd., Shizuoka, Japan) along with the instructions for sample collection. Subjects were requested to complete an online questionnaire to ask health conditions on the day of fecal sampling. The questions utilized for the analysis in this study were whether subjects had symptoms of diarrhea and/or fever, whether they received medications within one month, and their stool consistency measured by the Bristol Stool Scale (Supplementary Data 2).

### Food frequency questionnaire

Dietary habits were assessed using a previously validated brief-type, self-administered diet history questionnaire (BDHQ)^39,40^. Our study incorporated 70 food groups and 40 nutrients recommend for use because of their reliable values (http://www.ebnjapan.org/sitsumon/pdf/result/sample_20100507_aradata.pdf) (Supplementary Data 2). The values of nutrient and food intake were energy-adjusted using the density method (that is, amount per 1000 kcal of energy) to minimize the influence of dietary misreporting. In BDHQ, questions about demographics and physical characteristics were also asked, and these variables were used for the analysis (Supplementary Data 2).

### Lifestyle questionnaire

Each subject was asked to complete an online questionnaire about physical activity, smoking status, sleep duration, and stress response, and these variables were used for the analysis (Supplementary Data 2). To assess the subjects’ stress response, we used a subscale evaluated by 29 items of the Brief Job Stress Questionnaire (BJSQ)^41^. The total stress response consisted of 18 items related to the psychological stress response, including manifestations such as lassitude, irritation, fatigue, anxiety, and depression, and 11 items related to physical stress response to somatic symptoms. Higher scores were related to a higher stress response.

### Microbiological analyses

DNA extraction from fecal samples, subsequent sequencing of the V3-V4 region of bacterial 16S rRNA gene, and data processing of the sequences were conducted as described previously^15^. After acquiring the Illumina paired-end reads, the Bowtie-2 program^42^ (ver. 2–2.2.4) was used to remove reads mapped to the PhiX 174 sequence and the Genome Reference Consortium human build 38 (GRCh38). Thereafter, the 3’ region of each read with a PHRED quality score less than 17 was trimmed. Trimmed reads less than 150 bp in length with an average quality score less than 25 or those lacking paired reads were also removed. The trimmed paired-end reads were combined by the fastq-join script in EA-Utils^43^ (ver. 1.1.2–537). Potential chimeric sequences were removed by reference-based chimaera checking in USEARCH^44^ (ver. 5.2.32) and the Genomes OnLine Database (GOLD) (http://drive5.com/otupipe/gold.tz).

Non-chimeric sequences were analyzed via the QIIME software package version 1.8.0^45,46^. For genus-level analysis, the sequences were assigned to operational taxonomic units (OTUs) by open-reference OTU picking^47^ with a 97% pairwise identity threshold and the Greengenes reference database^48^.

DNA sequences corresponding to 16S rRNA gene data were deposited in DDBJ under accession numbers DRA007985-DRA007990 (Supplementary Data 15). For the relative abundances of genera, we normalized the values using the Box-Cox transformation using the function box.cox.powers in R (in the package ‘car’) and used the normalized values in subsequent analysis. Because our microbiome data included zero OTU read counts, we added the offset value, which was the minimum OTU read count across all the detected OTUs, to every OTU read count before the Box–Cox transformation.

### Effects of extrinsic and demographic variables

To investigate the associations of each of the 138 extrinsic and demographic variables with the relative abundances of genera or the alpha diversity, multivariable regression analyses were performed using the function lm in R and including sex and age variables as covariates. The 138 variables were classified into the following seven categories: (1) demographics, (2) physical characteristics, (3) nutrition, (4) food, (5) lifestyle, (6) stress response, and (7) health information. The nutrition variables and most of the food variables, which were equivalent to nutrient intake and food intake, respectively, were estimated from the BDHQ. The other variables were assessed by one of three questionnaires (screening survey questionnaire, lifestyle questionnaire, or health conditions questionnaire) or the BDHQ, as reported in Supplementary Data 2. The relative abundances of genera, alpha diversity indices, and each variable were used after *z*-score transformation. The statistical significance level was set to *P* < 3.62 × 10^−4^ based on multiple testing correction for the number of variables. The regression coefficients were visualized using the heatmap.2 function in R (in the package ‘gplots’), in which rows and columns were ordered based on a hierarchical clustering with the ward. D2 algorithm. The independence of the patterns of the two major genus clusters obtained from each hierarchical clustering based on association data of nutrition, food, and nondietary variables was evaluated by Fisher’s exact test using R. To investigate the association of each of the extrinsic and demographic variables with the beta diversity, non-metric multidimensional scaling (NMDS) was first performed using the metaMDS function in R (in the package ‘vegan’) for the weighted UniFrac metric data. Then, the associations between the variables and the NMDS ordination were analyzed using the envfit function in R (in the package ‘vegan’), and we found no variables with a significant association (*P* < 3.62 × 10^−4^).

### Bacterial association with host genetic variation

In the GWAS for the gut microbiota, extrinsic and demographic variables showing significant associations were incorporated as covariates in addition to the basic covariates (sex, age, and the first two principal components (PCs) from the PCA of the host genotypic data) in each GWAS. The number of PCs to be incorporated was determined based on examination of the screen plot of PC eigenvalues and assured by the genomic inflation factors close to 1. The first 20 PCs were subjected to inspection of the screen plot (Supplementary Fig. 7), and we found that the slope of the curve leveled off twice at two and five PCs, suggesting that either the first two or five PCs were relatively informative. When the first two PCs were used as covariates, the genomic inflation factors were equal to 1 for four of the five identified associations or close to 1 for the remaining association (1.01 for *Faecalibacterium*), demonstrating little inflation in *p*-values (Supplementary Fig. 8). Incorporation of the first five PCs did not change the genomic inflation factors. Taken together, we concluded that the first two PCs were sufficient to control population stratification in our analysis. The results of the GWASs were essentially unchanged when the first five PCs were incorporated. The variables used as covariates for GWASs are summarized in Supplementary Data 3 and 4, which are indicated in bold. To avoid overadjustment, the variable with the largest absolute value for a correlation coefficient among colinear variables (Spearman’s rank correlation coefficient > 0.7) was used as a representative. For the relative abundances of genera and the alpha diversity indices, we used a linear regression model implemented in PLINK version 1.9 under the assumption of additive effects of the alleles (0, 1, and 2). We tested the model fit for the association by comparing additive, dominant, and recessive models using linear regression. For the beta diversity index, the software package, microbiomeGWAS^49^, was used to test the associations. In addition to the analyses performed in combined male and female samples, sex-stratified analyses were also performed for each phenotype. For the five loci, we computed *p*-values testing for sex differences in the effect sizes as described previously^50^, and the statistical significance level was set to *P* < 0.01 based on multiple testing correction. Imputation of the missing genotypes was performed for 50 Mb regions surrounding the significantly associated loci by first pre-phasing using Eagle v2.4.1^51,52^ and then imputing using Minimac3^53^ with all samples in the 1000 Genomes Project Phase 3 (version 5)^54^ as a reference. For the purpose of quality control, we used imputed variants with an Rsq ≥ 0.7 in the subsequent GWASs. The proportion of variance explained by an individual SNP was calculated by 2 *f*(1–*f*) *a*^2^, where f is the frequency of an allele and a is its standardized coefficient^55^. SNPs were annotated with SNPnexus (http://snp-nexus.org/index.html)^56^, and the protein expression data of overlapping genes in intestinal and colon tissues were obtained according to The Human Protein Atlas (http://www.proteinatlas.org/)^28^. Manhattan plots and quantile-quantile (Q-Q) plots were generated using the qqman R package^57^, and the Q-Q plots for the five significant associations indicated no remarkable discrepancy from the null hypothesis (Supplementary Fig. 8). Regional association plots of SNPs were generated using LocusZoom 1.4^58^.

### Comparison with previously reported loci

For each of the 303 genome-wide suggestive SNPs found, we analyzed nearby SNPs previously reported to be associated with the microbiota parameters studied here^5,7,13,14^. We narrowed down the nearby candidate SNPs by a distance limit of 1 Mb, an overlap of the nearest genes. In the evaluation of the effects of previously reported loci, we chose 219 typing SNPs from previous reports^5,7,13,14^, which were reported as gut microbiota-associated SNPs or their tag SNPs (r2 ≥ 0.8 in HapMap JPT). The tag SNPs were selected using the --show-tags function in PLINK version 1.9. The statistical significance level was set to *P* < 2.28 × 10^−4^ based on multiple testing correction for the 219 SNPs analyzed.

### Evaluation of the cumulative effects of the GWAS-identified SNPs

The predictive power of a multiple regression model with the GWAS-identified SNPs was calculated. The SNPs were selected via the elastic net from genome-wide suggestive SNPs (results of sex-combined GWAS and sex-stratified GWAS) using the cv.glmnet function in R (in the package ‘glmnet’).

### SNP heritability estimation

SNP heritability was estimated by calculating the PVE by genome-wide SNPs using GEMMA v0.94^26^ on sex-combined data as previously described^7^ with some modification. Briefly, each of the Box-Cox transformed relative abundances of genera was regressed on the sex variable in advance, and then the residual was used for the PVE calculation. A genus was considered heritable if the SE of its PVE measurements did not intersect zero.

### Statistics and reproducibility

Software used for statistical calculation in this study: PLINK (https://www.cog-genomics.org/plink/), microbiomeGWAS (https://github.com/lsncibb/microbiomeGWAS), GEMMA (https://github.com/genetics-statistics/GEMMA), and R (https://www.r-project.org/). The statistical significance levels were set to *P* < 3.62 × 10^−4^ and *P* < 8.9 × 10^−8^ for the association analysis of the gut microbiota with the extrinsic and demographic variables and host genetic variants, respectively, which adjust for multiple testing correction for the number of variables or genetic variants per phenotype. Replication was not discussed in this study.

### Reporting summary

Further information on research design is available in the Nature Research Reporting Summary linked to this article.

## Supplementary information

Supplementary Figures

Description of Additional Supplementary Files

Supplementary Data 1

Supplementary Data 2

Supplementary Data 3

Supplementary Data 4

Supplementary Data 5

Supplementary Data 6

Supplementary Data 7

Supplementary Data 8

Supplementary Data 9

Supplementary Data 10

Supplementary Data 11

Supplementary Data 12

Supplementary Data 13

Supplementary Data 14

Supplementary Data 15

Reporting Summary

## Data Availability

DNA sequences corresponding to 16S rRNA gene data are available at the DNA Databank of Japan (DDBJ) under accession numbers DRA007985-DRA007990. GWAS Summary statistics for the microbiota parameters related to the five loci will be made publicly available from the National Human Genome Research Institute-European Bioinformatics Institute (NHGRI-EBI) GWAS Catalog, https://www.ebi.ac.uk/gwas/downloads/summary-statistics. The accession IDs are GCST90007008–GCST90007012. We also provide GWAS summary statistics for the relative abundances of the 21 core genera, which were conducted with imputed genetic data, under the accession IDs GCST90006987–GCST90007007. Host genetic data, which were derived from MYCODE, a personal genome service in Japan, cannot be shared publicly because their use, as per informed consent and Institutional Review Board approval, is restricted to MYCODE Research only.

## References

[1] Sommer F, Anderson JM, Bharti R, Raes J, Rosenstiel P (2017). The resilience of the intestinal microbiota influences health and disease. Nat. Rev. Microbiol..

[2] Singh RK (2017). Influence of diet on the gut microbiome and implications for human health. J. Transl. Med..

[3] Falony G (2016). Population-level analysis of gut microbiome variation. Science.

[4] Zhernakova A (2016). Population-based metagenomics analysis reveals markers for gut microbiome composition and diversity. Science.

[5] Rothschild D (2018). Environment dominates over host genetics in shaping human gut microbiota. Nature.

[6] Goodrich JK (2014). Human genetics shape the gut microbiome. Cell.

[7] Davenport ER (2015). Genome-wide association studies of the human gut microbiota. PLoS ONE.

[8] Goodrich JK (2016). Genetic determinants of the gut microbiome in UK twins. Cell Host Microbe.

[9] Turpin W (2016). Association of host genome with intestinal microbial composition in a large healthy cohort. Nat. Genet..

[10] Goodrich JK, Davenport ER, Clark AG, Ley RE (2017). The relationship between the human genome and microbiome comes into view. Annu. Rev. Genet..

[11] Lim MY (2017). The effect of heritability and host genetics on the gut microbiota and metabolic syndrome. Gut.

[12] Blekhman R (2015). Host genetic variation impacts microbiome composition across human body sites. Genome Biol..

[13] Wang J (2016). Genome-wide association analysis identifies variation in vitamin D receptor and other host factors influencing the gut microbiota. Nat. Genet..

[14] Bonder MJ (2016). The effect of host genetics on the gut microbiome. Nat. Genet..

[15] Kato K (2018). Association between functional lactase variants and a high abundance of Bifidobacterium in the gut of healthy Japanese people. PLoS ONE.

[16] Nishijima S (2016). The gut microbiome of healthy Japanese and its microbial and functional uniqueness. DNA Res..

[17] Haga H, Yamada R, Ohnishi Y, Nakamura Y, Tanaka T (2002). Gene based-SNP discovery as part of the Japanese Millennium Genome Project: identification of 190,562 genetic variations in the human genome. Single-nucleotide polymorphism. J. Hum. Genet..

[18] Akiyama M (2017). Genome-wide association study identifies 112 new loci for body mass index in the Japanese population. Nat. Genet..

[19] Imamura M (2016). Genome-wide association studies in the Japanese population identify seven novel loci for type 2 diabetes. Nat. Commun..

[20] O’Callaghan A, van Sinderen D (2016). Bifidobacteria and their role as members of the human gut microbiota. Front. Microbiol..

[21] Milani C (2016). Genomics of the genus Bifidobacterium reveals species-specific adaptation to the glycan-rich gut environment. Appl. Environ. Microbiol..

[22] Flak MB, Neves JF, Blumberg RS (2013). Immunology. Welcome to the microgenderome. Science.

[23] Dominianni C (2015). Sex, body mass index, and dietary fiber intake influence the human gut microbiome. PLoS ONE.

[24] Oki K (2016). Comprehensive analysis of the fecal microbiota of healthy Japanese adults reveals a new bacterial lineage associated with a phenotype characterized by a high frequency of bowel movements and a lean body type. BMC Microbiol..

[25] Suzuki Y (2017). Association between yogurt consumption and intestinal microbiota in healthy young adults differs by host gender. Front. Microbiol..

[26] Zhou X, Stephens M (2012). Genome-wide efficient mixed-model analysis for association studies. Nat. Genet..

[27] Uhlen M (2010). Towards a knowledge-based Human Protein Atlas. Nat. Biotechnol..

[28] Uhlén M (2015). Proteomics. Tissue-based map of the human proteome. Science.

[29] Hofer P (2017). Bayesian and frequentist analysis of an Austrian genome-wide association study of colorectal cancer and advanced adenomas. Oncotarget.

[30] Chen W, Liu F, Ling Z, Tong X, Xiang C (2012). Human intestinal lumen and mucosa-associated microbiota in patients with colorectal cancer. PLoS ONE.

[31] Zhu Q (2014). Analysis of the intestinal lumen microbiota in an animal model of colorectal cancer. PLoS ONE.

[32] Wu, M. et al. The dynamic changes of gut microbiota in *Muc2* deficient mice. *Int. J. Mol. Sci.* **19**, 10.3390/ijms19092809 (2018). 10.3390/ijms19092809 PMC6164417 30231491

[33] Awany D (2018). Host and microbiome genome-wide association studies: current state and challenges. Front. Genet..

[34] Bersaglieri T (2004). Genetic signatures of strong recent positive selection at the lactase gene. Am. J. Hum. Genet..

[35] Kurilshikov A, Wijmenga C, Fu J, Zhernakova A (2017). Host genetics and gut microbiome: challenges and perspectives. Trends Immunol..

[36] Wang J (2018). Meta-analysis of human genome-microbiome association studies: the MiBioGen consortium initiative. Microbiome.

[37] Purcell S (2007). PLINK: a tool set for whole-genome association and population-based linkage analyses. Am. J. Hum. Genet..

[38] Chang CC (2015). Second-generation PLINK: rising to the challenge of larger and richer datasets. Gigascience.

[39] Kobayashi S (2012). Both comprehensive and brief self-administered diet history questionnaires satisfactorily rank nutrient intakes in Japanese adults. J. Epidemiol..

[40] Kobayashi S (2011). Comparison of relative validity of food group intakes estimated by comprehensive and brief-type self-administered diet history questionnaires against 16 d dietary records in Japanese adults. Public Health Nutr..

[41] Ohno, Y., Shimomitsu, T., Nakamura, K. & Yokoyama, K. *Final Development of the Brief Job Stress Questionnaire Mainly Used for Assessment of the Individuals*. 126–164 (Japan, 2000).

[42] Langmead B, Salzberg SL (2012). Fast gapped-read alignment with Bowtie 2. Nat. Methods.

[43] Erik A (2013). Comparison of sequencing utility programs. Open Bioinform. J..

[44] Edgar RC, Haas BJ, Clemente JC, Quince C, Knight R (2011). UCHIME improves sensitivity and speed of chimera detection. Bioinformatics.

[45] Caporaso JG (2010). QIIME allows analysis of high-throughput community sequencing data. Nat. Methods.

[46] Kuczynski, J. et al. Using QIIME to analyze 16S rRNA gene sequences from microbial communities. *Curr Protoc Bioinform.* **27**, 1E-5 (2011). 10.1002/9780471729259.mc01e05s27 PMC4477843 23184592

[47] Rideout JR (2014). Subsampled open-reference clustering creates consistent, comprehensive OTU definitions and scales to billions of sequences. PeerJ.

[48] McDonald D (2012). An improved Greengenes taxonomy with explicit ranks for ecological and evolutionary analyses of bacteria and archaea. ISME J..

[49] Hua, X. et al. MicrobiomeGWAS: a tool for identifying host genetic variants associated with microbiome composition. 10.1101/031187 (2015). 10.3390/genes13071224 PMC9317577 35886007

[50] Randall JC (2013). Sex-stratified genome-wide association studies including 270,000 individuals show sexual dimorphism in genetic loci for anthropometric traits. PLoS Genet..

[51] Loh PR (2016). Reference-based phasing using the Haplotype Reference Consortium panel. Nat. Genet..

[52] Loh PR, Palamara PF, Price AL (2016). Fast and accurate long-range phasing in a UK Biobank cohort. Nat. Genet..

[53] Das S (2016). Next-generation genotype imputation service and methods. Nat. Genet..

[54] Auton A (2015). A global reference for human genetic variation. Nature.

[55] Gudbjartsson DF (2008). Many sequence variants affecting diversity of adult human height. Nat. Genet..

[56] Dayem Ullah AZ (2018). SNPnexus: assessing the functional relevance of genetic variation to facilitate the promise of precision medicine. Nucleic Acids Res..

[57] Turner, S. D. qqman: an R package for visualizing GWAS results using Q-Q and manhattan plots. *Journal of Open Source Software* **3**, 731 10.21105/joss.00731 (2014).

[58] Pruim RJ (2010). LocusZoom: regional visualization of genome-wide association scan results. Bioinformatics.

