# Supplementary material for: Genome-wide association studies and heritability analysis reveal the involvement of host genetics in the Japanese gut microbiota
Source: Commun Biol. 2020 Nov 18;3:686. doi: 10.1038/s42003-020-01416-z (PMC7674416; doi:10.1038/s42003-020-01416-z)
Supplement: Supplementary file 1 — Supplementary Figures [file 42003_2020_1416_MOESM1_ESM.pdf]

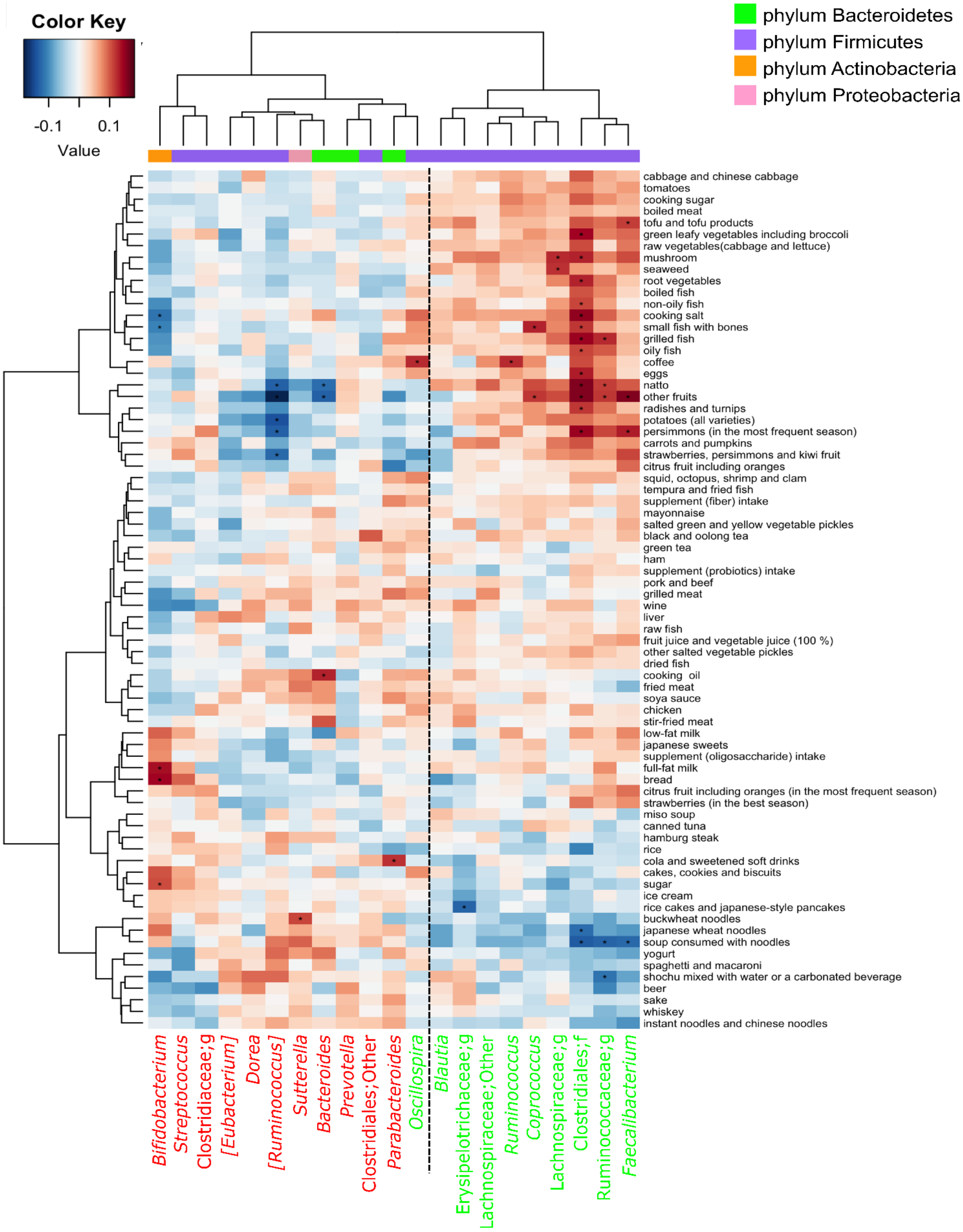

Supplementary Fig. 1. A hierarchical clustering of the 21 core genera based on association with food intake. Columns correspond to the 21 core genera in the Japanese gut; rows correspond to food intake. Red and blue denote positive and negative associations, respectively. The intensity of the colors represents the degree of association between the genus abundance and food intake as measured by a multiple linear regression analysis. Bacterial phyla are summarized by the color code on the top right. The dots indicate the associations that are significant after adjusting for multiple testing of 138 variables. Columns and rows are subjected to hierarchical clustering. The genera indicated in red and green are members of cluster I and cluster II, respectively, in Fig 1.

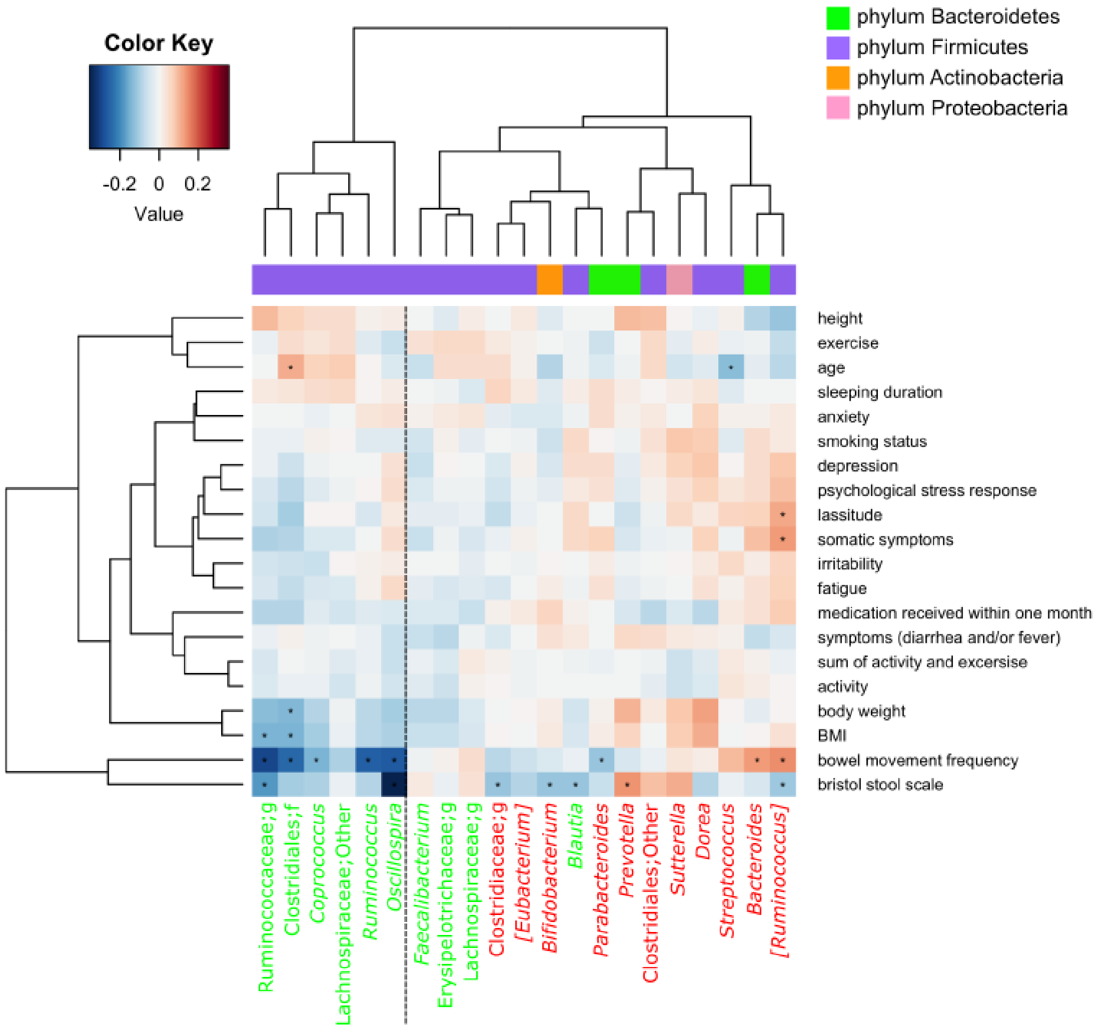

Supplementary Fig. 2. A hierarchical clustering of the 21 core genera based on association with nondietary variables. Columns correspond to the 21 core genera in the Japanese gut; rows correspond to nondietary variables. Red and blue denote positive and negative associations, respectively. The intensity of the colors represents the degree of association between the genus abundance and nondietary variables as measured by a multiple linear regression analysis. Bacterial phyla are summarized by the color code on the top right. The dots indicate the associations that are significant after adjusting for multiple testing of 138 variables. Columns and rows are subjected to hierarchical clustering. The genera indicated in red and green are members of cluster I and cluster II, respectively, in Fig 1.

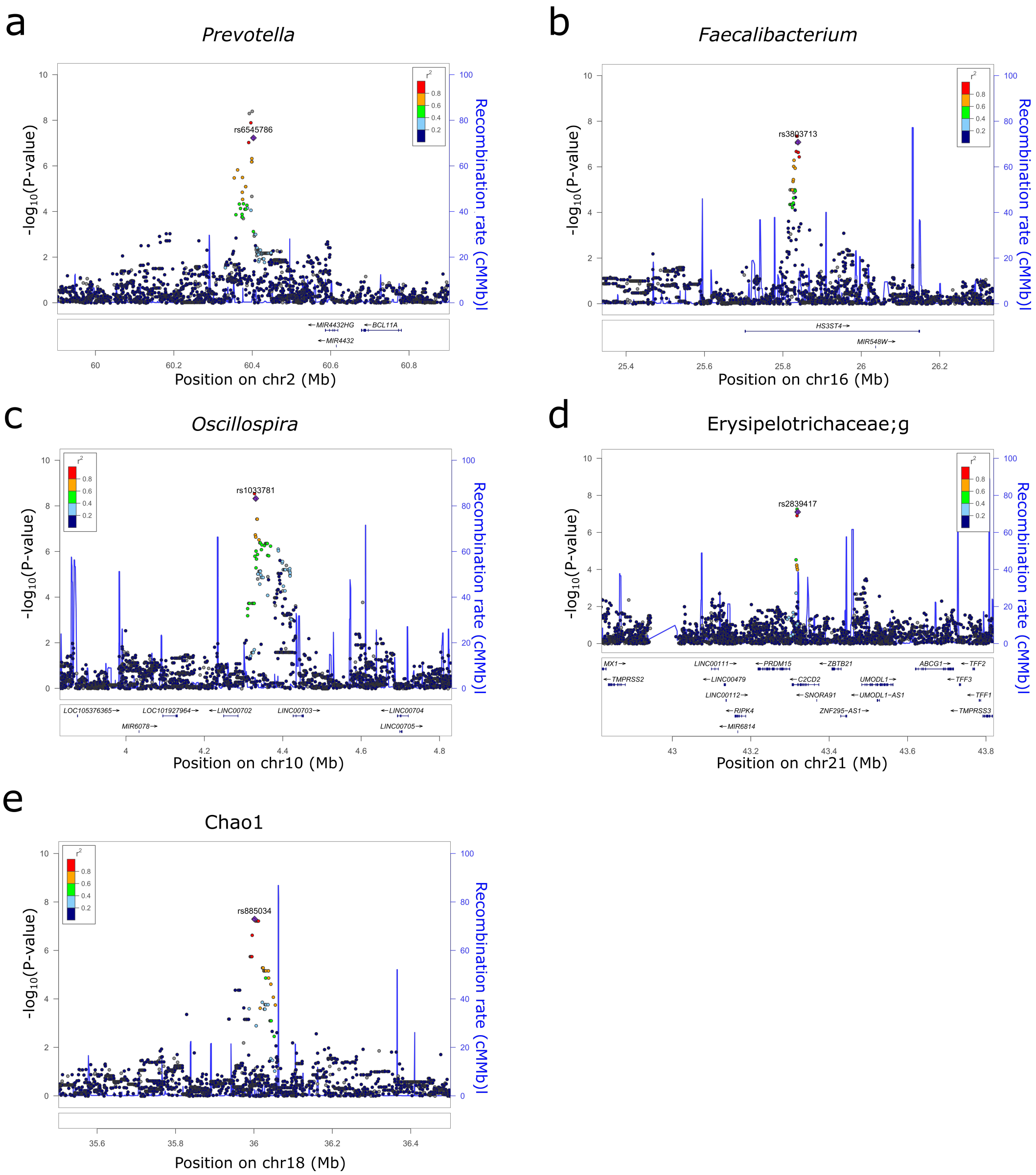

Supplementary Fig. 3. Regional association plots of novel genetic loci showing significant associations with the gut microbiota. (a-e) Regional association plots of each genome-wide SNP with respect to its chromosomal location (x-axis) and association -log10 p-values of SNPs; (left y-axis) for (a) ***Prevotella*** in males, (b) ***Faecalibacterium*** in males, (c) ***Oscillospira*** in females, (d) Erysipelotrichaceae;g in females, and (e) the diversity index Chao1 in females under the additive mod el for (a, b, d) and the dominant model for (c, e). SNPs are colored according to their degree of LD (r2) with the lead variant, which is represented by a purple diamond and labeled. The blue line shows the r ecombination rate (right y-axis). The lower part shows the RefSeq genes in the region. Coordinates ar e given according to human genome version hg19. The reference population for LD and recombination rate determination was HapMap hg19/1000 Genomes Nov. 2012 ASN. The Figure was generated using LocusZoom 1.4.

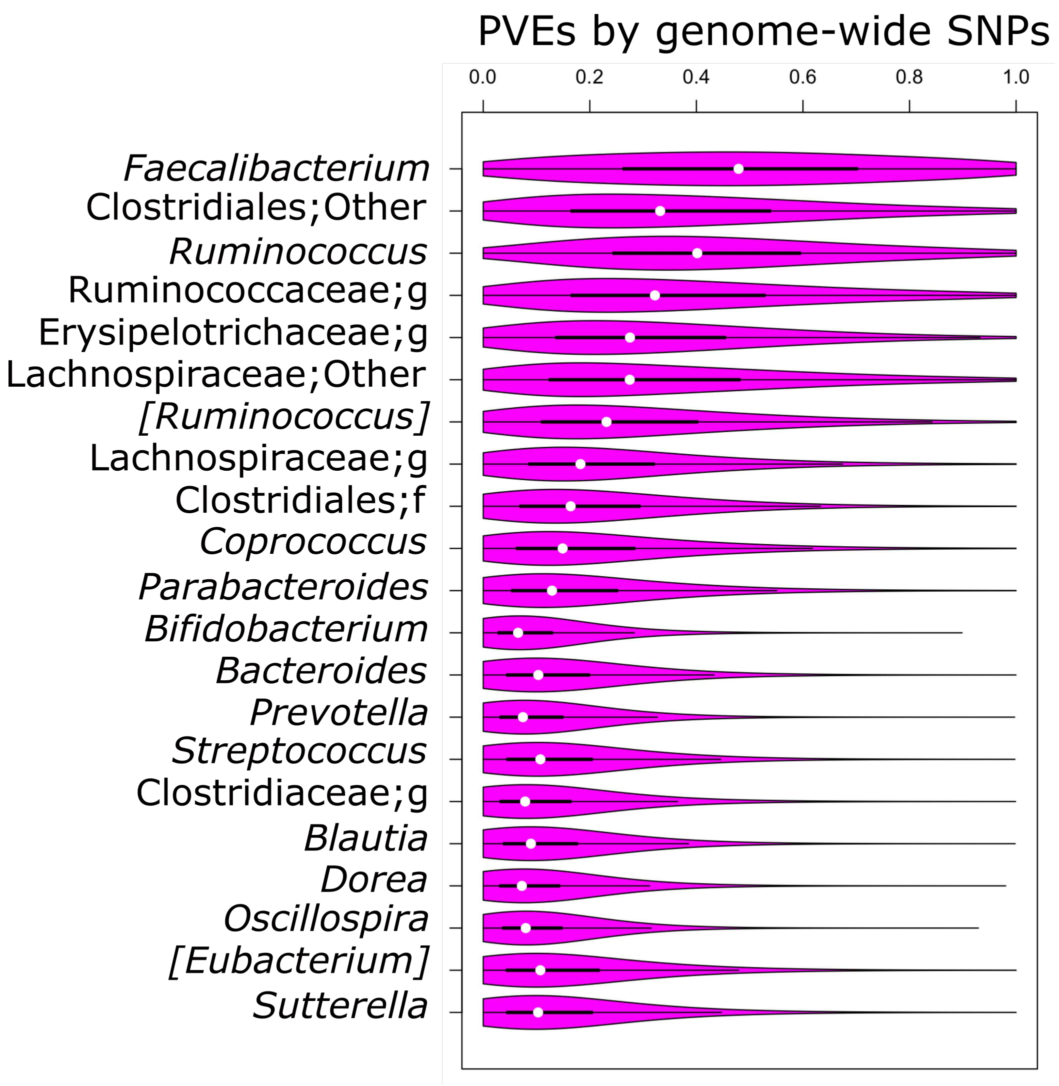

Supplementary Fig. 4. Violin plots of the posterior samples of PVE. The posterior samples were obtained using the Markov chain Monte Carlo-based method in GEMMA. The white dots represent the medians.

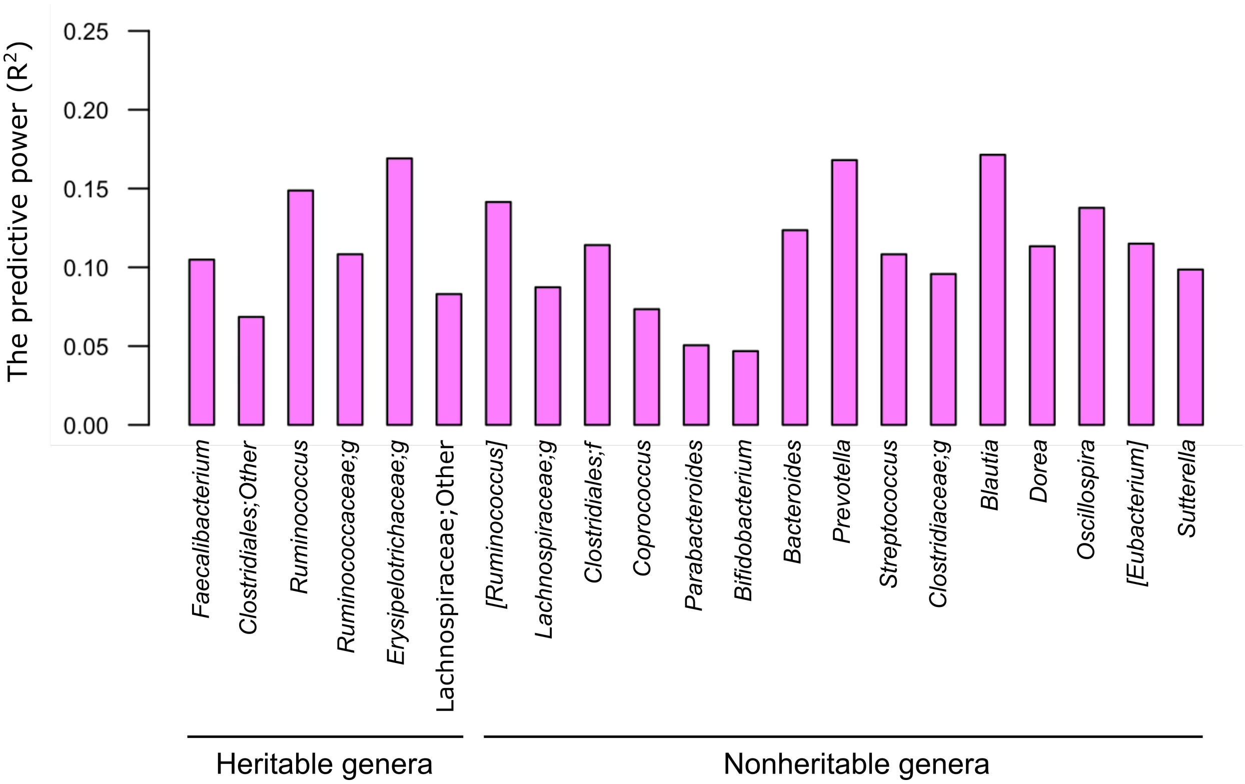

Supplementary Fig. 5. Cumulative contributions of the suggestively associated SNPs. The predictive power of a multiple regression model introducing the suggestively associated SNPs selected via the elastic net against the abundances of the 21 core genera. The 21 genera are arranged along the x-axis in descending order of the degree of SNP heritability, as shown in Fig 3.

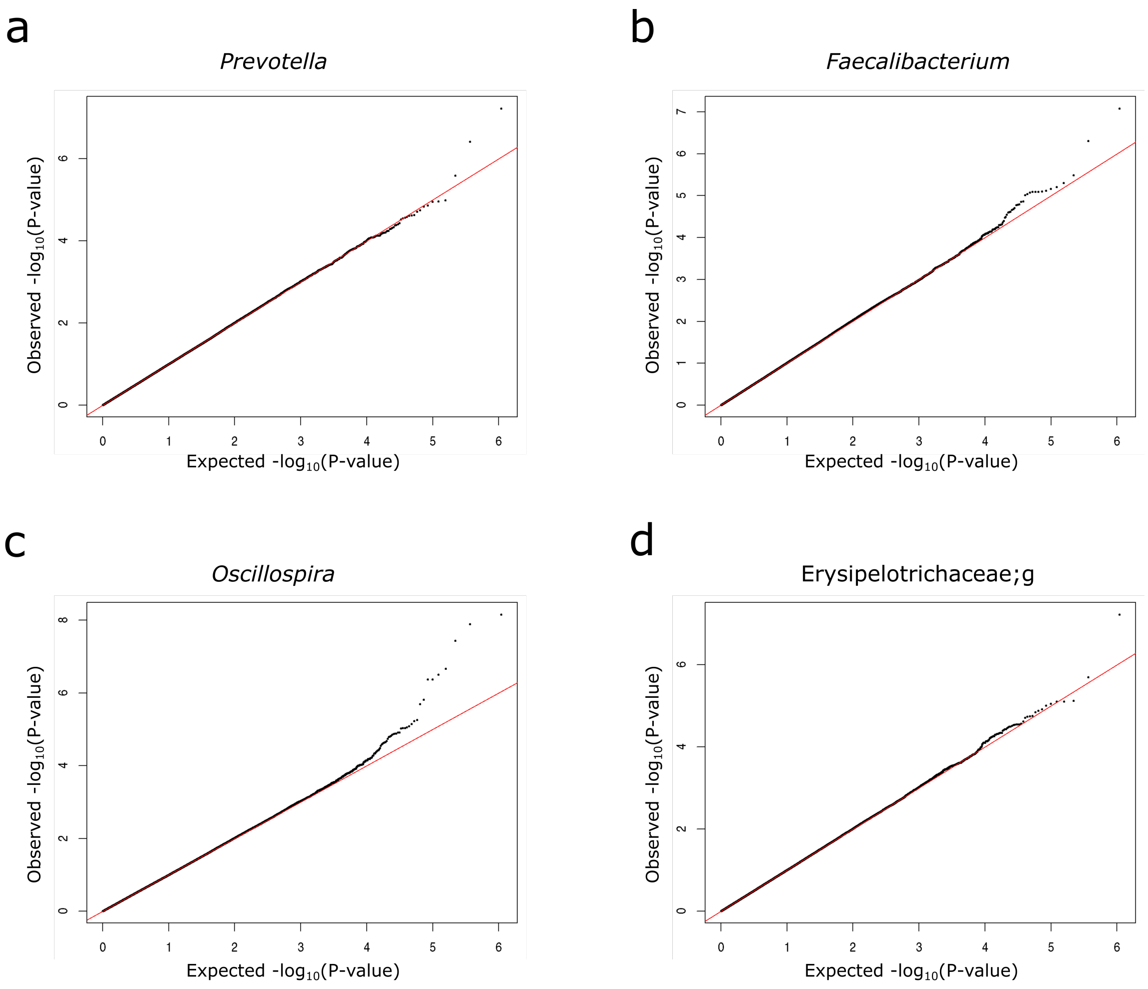

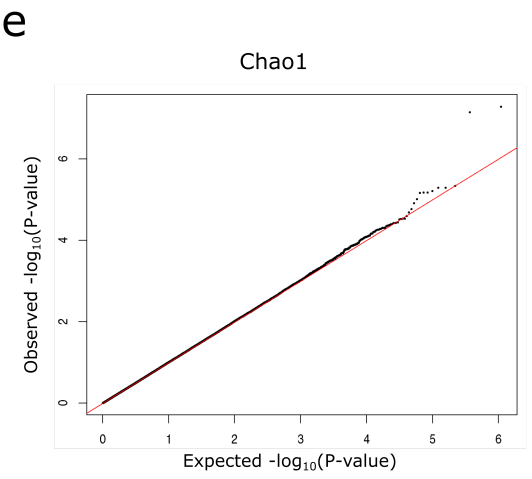

Supplementary Fig. 6. PCA of the subjects in our GWASs and in the Hapmap database. Two-dimensional plots of the first and the second principal components (PCs) obtained from PCA of the 1,068 subjects used in the microbiome GWASs (MYCODE) and HapMap data (ASW, African ancestry in Southwest USA; CEU, Utah residents with Northern and Western European ancestry from the CEPH collection; CHB, Han Chinese in Beijing, China; CHD, Chinese in Metropolitan Denver, Colorado; GIH, Gujarati Indians in Houston, Texas; JPT, Japanese in Tokyo, Japan; LWK, Luhya in Webuye, Kenya; MXL, Mexican ancestry in Los Angeles, California; MKK, Maasai in Kinyawa, Kenya; TSI, Toscani in Italia; YRI, Yoruba in Ibadan, Nigeria).

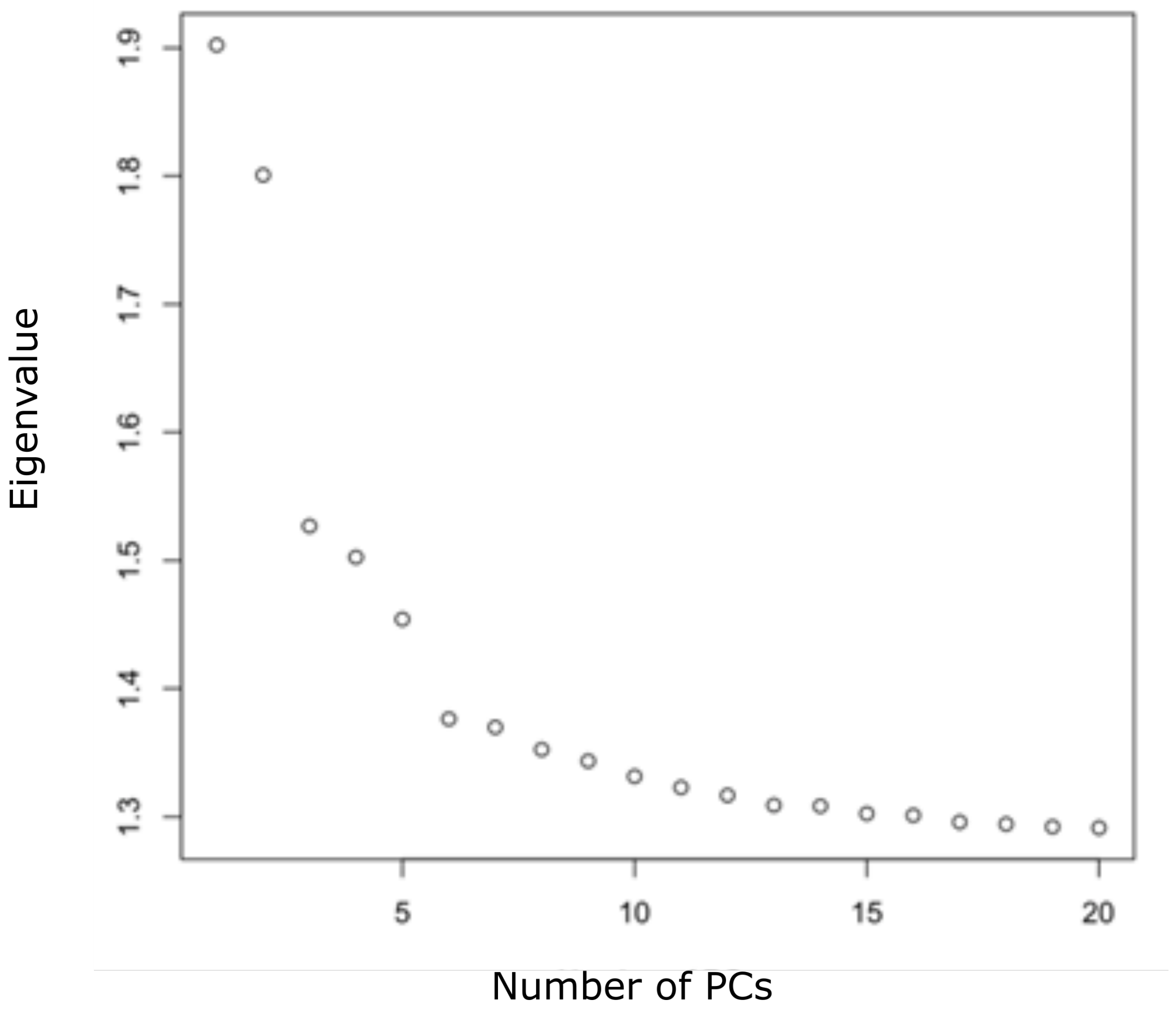

Supplementary Fig. 7. Screen plot of the first 20 PCs from the PCA of the host genotypic data. Eigenvalue of each principal component (PC), which is obtained from the PCA of the host genotypic data, is plotted.

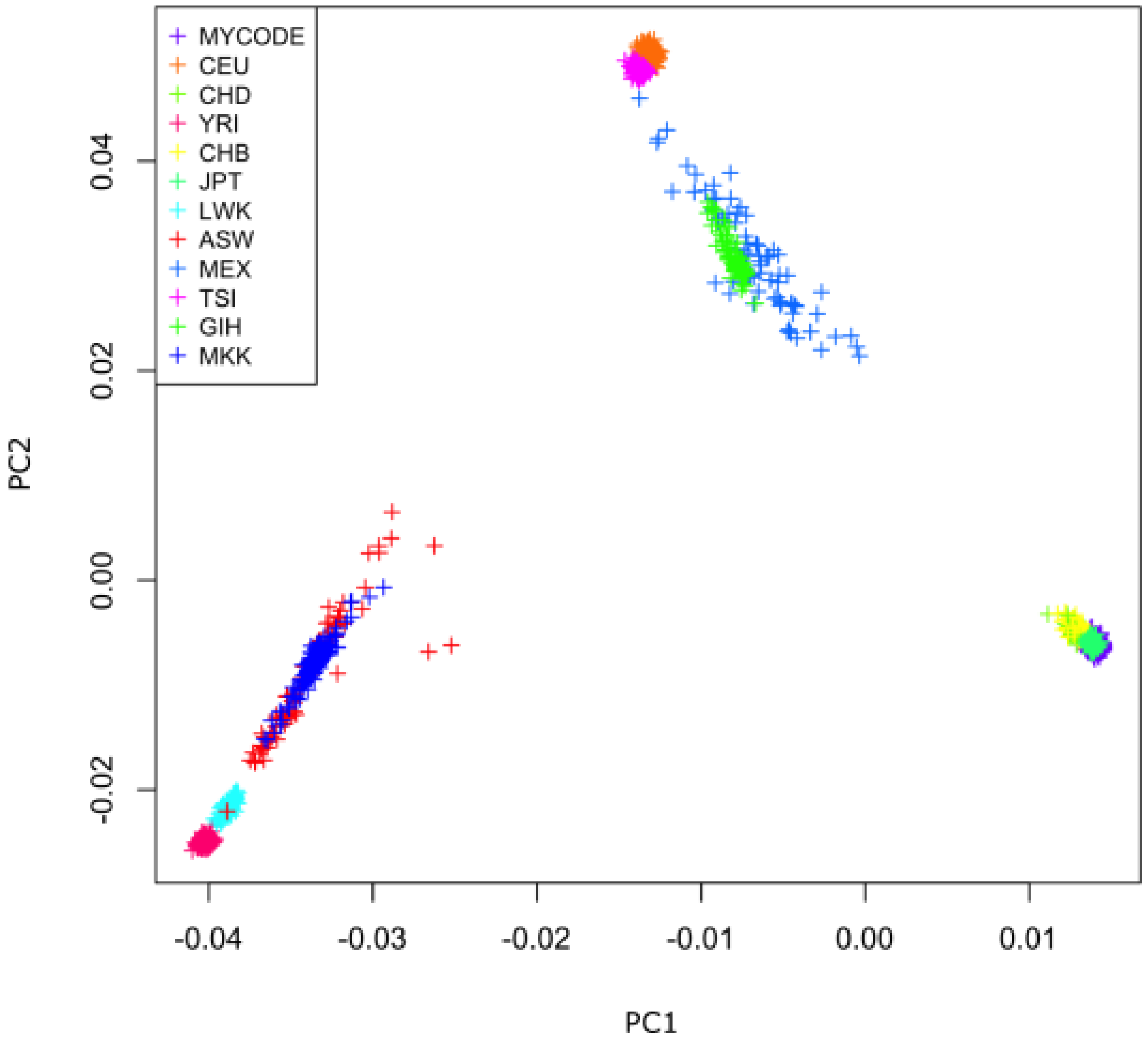

Supplementary Fig. 8. Q-Q plots of novel associations. (a-e) Q-Q plots for (a) ***Prevotella*** in males, (b) ***Faecalibacterium*** in males, (c) ***Oscillospira*** in females, (d) Erysipelotrichaceae;g in females, and (e) the diversity index Chao1 in females under the additive model for (a, b, d) and the dominant model for (c ,e).
