# Supplementary material for: Genome-wide association studies and heritability analysis reveal the involvement of host genetics in the Japanese gut microbiota
Source: Commun Biol. 2020 Nov 18;3:686. doi: 10.1038/s42003-020-01416-z (PMC7674416; doi:10.1038/s42003-020-01416-z)
Supplement: Supplementary file 2 — Description of Additional Supplementary Files [file 42003_2020_1416_MOESM2_ESM.pdf]

Description of additional supplementary files

File name: Supplementary Data 1.

Description: The relative abundances of the 21 core genera and the alpha diversity indices

File name: Supplementary Data 2.

Description: Variables used in the correlation analysis

File name: Supplementary Data 3.

Description: The significant associations between the relative abundances of bacterial groups and extrinsic and demographic variables

File name: Supplementary Data 4.

Description: The significant associations between the alpha diversity indices and extrinsic and demographic variables

File name: Supplementary Data 5.

Description: The associations between the relative abundances of bacterial groups and nutrition variables

File name: Supplementary Data 6.

Description: Genome-wide suggestive SNPs associated with the microbiota parameters in sex-combined analysis

File name: Supplementary Data 7.

Description: Genome-wide suggestive SNPs associated with the microbiota parameters in sex-stratified analysis

File name: Supplementary Data 8.

Description: Sex differences in the significantly associated SNPs

File name: Supplementary Data 9.

Description: Sex differences in the significantly associated SNPs

File name: Supplementary Data 10.

Description: SNPs reported to be associated with the microbiota in previous studies

File name: Supplementary Data 11.

Description: GWAS results of SNPs reported to be associated with the microbiota in previous studies

File name: Supplementary Data 12.

Description: SNP heritability of the microbiota parameters

File name: Supplementary Data 13.

Description: Cumulative contributions of GWAS-identified genetic factors

File name: Supplementary Data 14.

Description: SNP heritability estimation of the heritable taxa reported previously

File name: Supplementary Data 15.

Description: Fecal sample information
