# Supplementary material for: Genome-wide association studies and heritability analysis reveal the involvement of host genetics in the Japanese gut microbiota
Source: Commun Biol. 2020 Nov 18;3:686. doi: 10.1038/s42003-020-01416-z (PMC7674416; doi:10.1038/s42003-020-01416-z)
Supplement: Supplementary file 18 — Reporting Summary [file 42003_2020_1416_MOESM18_ESM.pdf]

nature research

Corresponding author(s): Sachiko Ishida

Last updated by author(s): 2020.09.18

Reporting Summary

Nature Research wishes to improve the reproducibility of the work that we publish. This form provides structure for consistency and transparency in reporting. For further information on Nature Research policies, see Authors & Referees and the Editorial Policy Checklist .

Statistics

For all statistical analyses, confirm that the following items are present in the figure legend, table legend, main text, or Methods section.

n/a	Confirmed	
☐	☒	The exact sample size (*n*) for each experimental group/condition, given as a discrete number and unit of measurement
☐	☒	A statement on whether measurements were taken from distinct samples or whether the same sample was measured repeatedly
☐	☒	The statistical test(s) used AND whether they are one- or two-sided<br>*Only common tests should be described solely by name; describe more complex techniques in the Methods section.*
☐	☒	A description of all covariates tested
☐	☒	A description of any assumptions or corrections, such as tests of normality and adjustment for multiple comparisons
☐	☒	A full description of the statistical parameters including central tendency (e.g. means) or other basic estimates (e.g. regression coefficient) AND variation (e.g. standard deviation) or associated estimates of uncertainty (e.g. confidence intervals)
☐	☒	For null hypothesis testing, the test statistic (e.g. *F*, *t*, *r*) with confidence intervals, effect sizes, degrees of freedom and *P* value noted<br>*Give P values as exact values whenever suitable.*
☒	☐	For Bayesian analysis, information on the choice of priors and Markov chain Monte Carlo settings
☒	☐	For hierarchical and complex designs, identification of the appropriate level for tests and full reporting of outcomes
☐	☒	Estimates of effect sizes (e.g. Cohen's *d*, Pearson's *r*), indicating how they were calculated

Our web collection on statistics for biologists contains articles on many of the points above.

Software and code

Policy information about availability of computer code

Data collection	No software tool is involved in data collection
Data analysis	Unix platform based tools: PLINK, GEMMA, microbiomeGWAS, Eagle (v2) , Minimac3, Bowtie-2, EA-Utils, USEARCH, QIIME<br>Analysis platform: R, Python<br>No custom code is in use

For manuscripts utilizing custom algorithms or software that are central to the research but not yet described in published literature, software must be made available to editors/reviewers. We strongly encourage code deposition in a community repository (e.g. GitHub). See the Nature Research guidelines for submitting code & software for further information.

Data

Policy information about availability of data

All manuscripts must include a data availability statement. This statement should provide the following information, where applicable:

- Accession codes, unique identifiers, or web links for publicly available datasets
- A list of figures that have associated raw data
- A description of any restrictions on data availability

DNA sequences corresponding to 16S rRNA gene data were deposited in DDBJ under accession numbers DRA007985-DRA007990. GWAS Summary statistics for the microbiota parameters related to the five loci will be made publicly available from the National Human Genome Research Institute-European Bioinformatics Institute (NHGRI-EBI) GWAS Catalog, https://www.ebi.ac.uk/gwas/downloads/summary-statistics. The accession IDs are GCST90007008~GCST90007012. We also provide GWAS summary statistics for the relative abundances of the 21 core genera, which were conducted with imputed genetic data, under the accession IDs GCST90006987~GCST90007007. Host genetic data, which were derived from MYCODE, a personal genome service in Japan, cannot be shared publicly because their use, as per informed consent and Institutional Review Board approval, is restricted to MYCODE Research only.

Field-specific reporting

Please select the one below that is the best fit for your research. If you are not sure, read the appropriate sections before making your selection.

- [x] Life sciences
- [ ] Behavioural & social sciences
- [ ] Ecological, evolutionary & environmental sciences

For a reference copy of the document with all sections, see nature.com/documents/nr-reporting-summary-flat.pdf

Life sciences study design

All studies must disclose on these points even when the disclosure is negative.

Sample size	1,068 healthy Japanese adults<br>We determined the sample size using two previous studies as reference. In the two studies, GWASs for microbiota (Blekhmanet al., Genome Biology, 2015; Bonder MJ et al., Nat Genet. 2016) were conducted using less than 1,000 individuals and discovered susceptible loci.
Data exclusions	Of 1,250 participants enrolled in this study, a total of 182 participants were removed from data analysis due to refusal to participate (n = 64), pregnancy or lactation (n = 7), medication use within the last two weeks (n = 96), and mismatch with the criteria for a standard quality control protocol for GWAS (n = 15).
Replication	No immediate replication is discussed.
Randomization	Randomization is not relevant to our study design.
Blinding	Blinding is not relevant to our study design.

Reporting for specific materials, systems and methods

We require information from authors about some types of materials, experimental systems and methods used in many studies. Here, indicate whether each material, system or method listed is relevant to your study. If you are not sure if a list item applies to your research, read the appropriate section before selecting a response.

Materials & experimental systems

n/a	Involved in the study	
x		Antibodies
x		Eukaryotic cell lines
x		Palaeontology
x		Animals and other organisms
	x	Human research participants
x		Clinical data

Methods

n/a	Involved in the study	
x		ChIP-seq
x		Flow cytometry
x		MRI-based neuroimaging

Human research participants

Policy information about studies involving human research participants

Population characteristics	Subjects (527 male; median age: 41 years) were recruited from the customer base of MYCODE, a personal genome service in Japan.<br>Subjects were genotyped on the Infinium OmniExpress-24+ BeadChip or Human OmniExpress-24+ BeadChip (Illumina Inc., San Diego, CA, United States). All of these experiments were performed at the DLS laboratory.
Recruitment	After purchase of MYCODE, a personal genome service in Japan, the customers sent back their saliva samples for genetic testing to the DeNA Life Science's (DLS's) laboratory with a written application form. On the application form, they can choose whether to give consent to participate in MYCODE Research, in which their anonymized genetic data and/or health-related information would be used for scientific research purposes.<br>Among participants in MYCODE Research, people between 20 and 64 years old were invited to participate in the specific research for the present study by an email informing its overview. Potential participants expressing their interest provided informed consent for this study and completed a screening questionnaire online.
Ethics oversight	The ethics committee of DLS Inc. (protocol #20160727_1) and the ethical committee of Institute of Medical Science, the University of Tokyo (Tokyo, Japan) (IMSUT-IRB) (protocol #29-29-1125)

Note that full information on the approval of the study protocol must also be provided in the manuscript.
